# Unilateral focused ultrasound thalamotomy for tremor-dominant Parkinson’s disease: blinded evaluation and imaging correlation

**DOI:** 10.1093/braincomms/fcaf303

**Published:** 2025-08-19

**Authors:** James Peters, Joel Maamary, Kain Kyle, Isabelle Osborne, Duncan Wilson, Lyndsey Jones, Sam Bolitho, Michael Barnett, Chenyu Wang, Yael Barnett, Benjamin Jonker, Stephen Tisch

**Affiliations:** Department of Neurology, St Vincent’s Health Network, Darlinghurst, New South Wales 2010, Australia; School of Medical Sciences, The University of New South Wales, Darlinghurst, New South Wales 2010, Australia; Department of Neurology, St Vincent’s Health Network, Darlinghurst, New South Wales 2010, Australia; School of Medical Sciences, The University of New South Wales, Darlinghurst, New South Wales 2010, Australia; Sydney Neuroimaging Analysis Centre, Brain and Mind Centre, Camperdown, New South Wales 2050, Australia; Department of Neurology, St Vincent’s Health Network, Darlinghurst, New South Wales 2010, Australia; Department of Neurology, Westmead Hospital, Westmead, New South Wales 2145, Australia; Department of Neurology, St Vincent’s Health Network, Darlinghurst, New South Wales 2010, Australia; Department of Neurology, St Vincent’s Health Network, Darlinghurst, New South Wales 2010, Australia; Sydney Neuroimaging Analysis Centre, Brain and Mind Centre, Camperdown, New South Wales 2050, Australia; Department of Neurology, Royal Prince Alfred Hospital, Camperdown, New South Wales 2050, Australia; Sydney Neuroimaging Analysis Centre, Brain and Mind Centre, Camperdown, New South Wales 2050, Australia; School of Medical Sciences, The University of New South Wales, Darlinghurst, New South Wales 2010, Australia; Department of Radiology, St Vincent’s Health Network, Darlinghurst, New South Wales 2010, Australia; School of Medical Sciences, The University of New South Wales, Darlinghurst, New South Wales 2010, Australia; Department of Neurosurgery, St Vincent’s Health Network, Darlinghurst, New South Wales 2010, Australia; Department of Neurology, St Vincent’s Health Network, Darlinghurst, New South Wales 2010, Australia; School of Medical Sciences, The University of New South Wales, Darlinghurst, New South Wales 2010, Australia

**Keywords:** Parkinson’s disease, tremor, thalamotomy, focused ultrasound

## Abstract

There have been promising outcomes from the use of unilateral High-intensity focused ultrasound (HiFUS) thalamotomy in tremor-dominant Parkinson’s disease. However, the reliability of this treatment has been questioned due to the high rate of tremor relapse. Authors have hypothesized that treatment failure is due to insufficient HiFUS lesion size, though detailed volumetric lesion analyses are lacking. To report the blinded tremor outcomes of unilateral HiFUS thalamotomy in tremor-dominant Parkinson’s disease and correlate these outcomes with lesion characteristics, including the dentatorubrothalamic tract ablation overlap, which may provide valuable insights into the mechanisms behind tremor relapse and ultimately refine the optimal HiFUS target for tremor in Parkinson’s disease. Retrospective review of consecutively treated tremor-dominant Parkinson’s disease patients followed under a uniform protocol. Blinded tremor analysis was completed on pre- and post-operative videos. Patients were classified into two groups: ‘responder’ (≥50% improvement in Hand Tremor Score) or ‘suboptimal responder’ (<50% improvement in Hand Tremor Score) at the last follow-up. 17 patients with tremor-dominant Parkinson’s disease underwent a unilateral HiFUS thalamotomy at our centre. Pre- and post-operative videos were available in 15 patients for analysis. Baseline median Hand Tremor Score was 11.0 (9.5–14.5), improving to 6.0 (1–13.5) over a median 24-month (3–36) follow-up period (*P* = 0.098). Seven patients had ≥50% improvement in Hand Tremor Score, while eight patients had <50% improvement in Hand Tremor Score at the last follow-up. At the final follow-up, the median change in Hand Tremor Score from baseline was 91% in responders, compared to an 8% increase in the suboptimal responders (*P* < 0.002). Levodopa daily dose equivalent increased by 17% in responders (*P* = 0.043), and the difference between responders and suboptimal responders was significant at the final follow-up (*P* = 0.024). There was a trend for larger lesions in the suboptimal responders, 157.5mm^3^ (113.4–215) compared to 119.7mm^3^ (111.6–237.6) in responders. Further, the dentatorubrothalamic core lesion overlap was greater in the suboptimal responders, 41.7% (40.5–48.8%), compared to the responders, 27.1% (13.8–39.3%), (*P* = 0.010), and was associated with a higher Hand Tremor Score at the final follow-up. We found that unilateral HiFUS thalamotomy in tremor-dominant Parkinson’s Disease resulted in sustained tremor reduction in approximately 50% of patients but was also in the context of higher levodopa replacement. These favourable outcomes did not correlate with DRTT ablation overlap or lesion size, providing indirect evidence that the most efficacious HiFUS thalamic tremor target differs between essential tremor and tremor-dominant Parkinson’s Disease.

## Teaser

Peters *et al*. report variable outcomes following unilateral high-intensity focused ultrasound thalamotomy in tremor-dominant Parkinson’s disease. Contrary to previous assumptions, favourable outcomes did not correlate with either dentatorubrothalamic tract ablation overlap or lesion size. These findings may help refine the optimal high-intensity focused ultrasound target in tremor-dominant Parkinson’s disease.

## Graphical Abstract

**Graphical Abstract:**
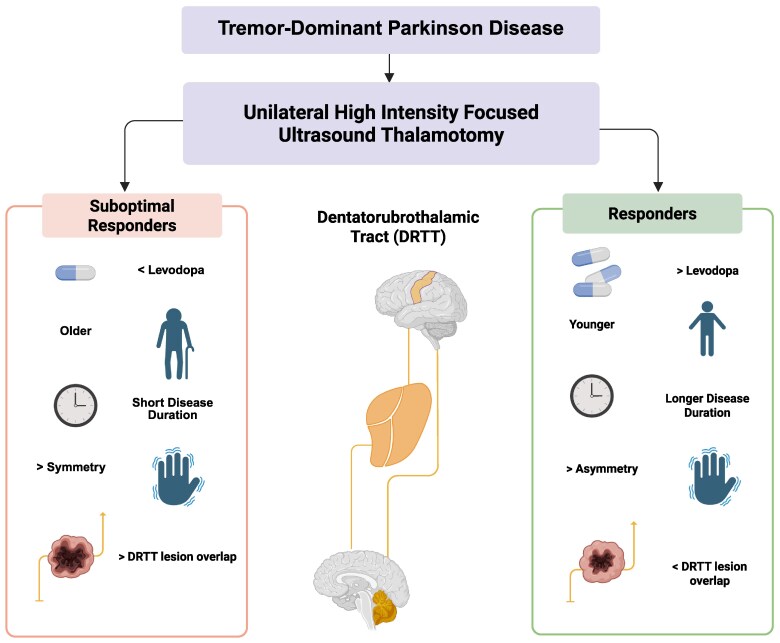

## Introduction

High-intensity focused ultrasound (HiFUS) is an incisionless, minimally invasive technique that utilizes ultrasonic energy to create thermal lesions.^1^ In movement disorders, the modality of treatment is ideal for patients who are not candidates for traditional stereotactic neurosurgical procedures or wish to avoid an open operation and/or insertion of permanent hardware. In medication-refractory tremor-dominant Parkinson’s Disease (TdPD), the promising outcomes from a HiFUS sham-control randomized clinical trial targeting the thalamus ^2^ led to the United States Food and Drug Administration (FDA) approval of the MRgFUS system for the treatment of TdPD in 2018. However, compared to the more common indication, essential tremor (ET),^3^ there is a relative lack of post-marketing evidence for HiFUS treatment in TdPD, and the treatment is currently not endorsed by key international societies outside of clinical trials or registries.^4^

A number of groups have published favourable outcomes following *Bond and colleagues* pivotal trial.^5-9^ These studies all involve unblinded assessments and have high attrition rates of follow-up beyond 12 months. There is a general concern within the movement disorders community that the effect of a unilateral thalamic HiFUS treatment for TdPD is not durable, with tremor relapses reported in the short (<6 months), medium (6–12 months) and long-term (>12 months).^8-11^ Treatment failure in TdPD is poorly understood; recently, it has been suggested to be associated with younger patients and insufficient lesion size, while others have attributed it to inappropriate thalamic targeting.^7,9,12,13^

The optimal site for a unilateral HiFUS thalamotomy in ET is generally considered to be at the posterior region of the ventral intermedius nucleus (Vim) of the thalamus. While several studies have also correlated the clinical outcome with the proportion of dentatorubrothalamic tract (DRTT) lesion overlap.^14-17^ The latter aligns with the notion that the optimal stereotactic target for tremor is a brain circuit with pathological oscillatory activity. In ET, the DRTT is the implicated circuit, coursing the motor cortex, dentate nucleus, thalamus and back to the motor cortex.^18^ While in Parkinson’s Disease (PD), dopamine denervation of the basal ganglia (BG) is a prerequisite for tremor with subsequent influence on the DRTT, directly and indirectly via the primary motor cortex.^19,20^ Despite these pathological differences, the HiFUS target for TdPD has, in large been inferred from the ET literature, the ‘traditional’ VIM target.^12^

Using blinded video assessments, treatment parameters and lesion characteristics, we have retrospectively analysed the clinical outcomes of patients with TdPD who were consecutively treated with a unilateral HiFUS treatment targeting the thalamus and prospectively followed under a uniform protocol at St Vincent’s Hospital, Sydney.

## Materials and methods

Seventeen patients underwent a unilateral HiFUS thalamotomy for medication-refractory TdPD between March 2019 and February 2023 at St Vincent’s Hospital, Sydney. All patients were enrolled in the prospective study, ‘Capturing outcomes in MRgFUS interventions for tremor.’ Clinical assessments were performed at baseline and at 1-, 3-, 6-, 12-, 24-, and 36-month time points. These included video assessments, Unified Parkinson’s Disease Rating Scale (UPDRS-III), Parkinson’s Disease Questionnaire (PDQ-39) summary index^21^ and levodopa equivalent daily dose (LEDD). Patients were assessed *on* medication, having taken their usual PD medications on the day of assessment, to demonstrate the medication-refractory nature of the tremor, the method adopted by Bond and colleagues.^2^

Recording of adverse events (AEs) was performed using a standardized template adapted from previous published MRgFUS studies.^22^ AEs were graded in accordance with Common Terminology Criteria for Adverse Events, version 5.0. For example, if the subject reported a decline in balance and/or walking after the procedure without objective evidence on examination, this was recorded as a Grade 1 gait disturbance. Grade 2 gait disturbance required objective evidence on clinical examination, with minor impairment of function not requiring assistance, and Grade 3 gait disturbance AE also required objective evidence on clinical examination, but at least moderate impairment of function, requiring assistance. This gradation scaling was used for all AE’s.

### Standard protocol approvals, registrations, and patient consent

The study was conducted in accordance with the Helsinki Declaration on human experimentation (revised 2013) and approved by the St Vincent’s Hospital Research Ethics Committees (ETH00670). All participants provided written informed consent.

### Blinded tremor evaluation

Baseline and post-HiFUS patient videos were recorded by a research nurse (L.J.). Patients wore a red disposable surgical head cap to ensure raters were blinded. Baseline and last follow-up assessments were randomized (J.P.) and presented to expert clinicians (I.O., S.T.) to perform the tremor evaluation in their own time. Evaluation was performed using part A and B of the Clinical Rating Scale for Tremor (CRST),^23^ enabling the blinded calculation of the treated (HTS) and untreated hand tremor scores (HuTS).^22^

### Focused ultrasound thalamotomy

The details of the HiFUS procedure performed by the neurosurgeon (B.J.) at our institution have previously been published and currently do not vary between tremor subtypes.^10,24^ In short, the Vim nucleus of the thalamus was targeted in all patients using traditional stereotactic planning. The anteroposterior (Y) coordinate was identified to be located at, or slightly anterior to, a point that is one-quarter of the total anterior commissure–posterior commissure (AC–PC) distance from the PC. Laterality (X) was determined to be approximately 14 mm from the midline, or 10–12 mm from the lateral wall of the third ventricle. Verticality (Z) was determined to be at the level of the AC–PC plane. Additional sonication in the posterior subthalamic area (PSA) occurred in patients who, despite at least three therapeutic treatments (maximal average temperature >53° for 3 s), had persistent clinically significant tremor. The PSA lesion was performed by targeting the white matter equidistant between the medial border of the subthalamic nucleus and the lateral border of the red nucleus at its equator, corresponding to coordinates of approximately, Y = 6 mm anterior to the PC, X = 9.5 mm, and Z = 3.5 mm below AC–PC.^14^

### MRI analysis

Magnetic resonance imaging (MRI) was acquired 1–7 days prior to treatment and again on day 1. Pre-treatment imaging was acquired on a 3-T (MRI) scanner (SIGNA Architect, General Electric, Milwaukee, WI) and included a sagittal three-dimensional (3D) Inversion Recovery prepared Fast Spoiled Gradient Recalled T1-weight image (T1-WI) and multi-shell diffusion MRI (dMRI). Post-treatment imaging was acquired on a 3-T Philips Ingenia (Philips Inc., Best, the Netherlands) and included axial 3D Inversion Recovery Fast Field Echo T1-WI.

To determine the HiFUS ablation volume, the post-treatment T1-WI was linearly aligned to the pre-treatment T1-WI using FLIRT in FSL. The ablated region was segmented manually by a trained neuroimaging analyst using ITK-SNAP. The hypointense core (zone 1) and hyperintense rim (zone 2) of the ablation were individually segmented. The pre-treatment dMRI data were processed to reconstruct the DRTT using the default MRtrix pipeline for probabilistic fibre tracking.^25^ Seeding in the dentate nucleus, with inclusion masks in the contralateral red nucleus, thalamus and pre-central cortex.^26^ Fibre tracking was terminated after 3000 streamlines satisfied the inclusion criteria, and then reduced to the 1000 most coherent streamlines with DIPY.^27^

The overlap of the ablations with the DRTT was assessed by calculating the fraction of streamlines that traversed either zone 1 or zone 2 of the ablation volumes, which are presented as a percentage. In cases that required an additional PSA lesion, only streamlines that were unique to the PSA ablation and did not traverse the VIM ablation were incorporated in the additional analysis to avoid duplication.

BrainLab software was used to determine the stereotactic atlas-based location (X, Y, and Z) of the HiFUS ablations in reference to the PC. The preoperative T1-W1 image was fused with the day-1 post-operative T1–W1 image. The AC–PC was defined on the preoperative image. The centre of the ablation was identified in the sagittal, coronal and axial planes. Further, the Y coordinate was also identified as a percentage to account for differences in the AC–PC distance.

### Statistical analysis

The cohort was categorized into ‘Responders’ (R), equal or greater than 50% improvement in blinded HTS at the last follow-up, and ‘Suboptimal Responders’ (SR), less than 50% improvement in blinded HTS at the last follow-up. The 50% dichotomy cut-off was pre-specified and was chosen as it aligned with *Bond and colleagues’* definition of ‘success’.^2^ Student T-test or Mann-Whitney test for normal and non-normally distributed data were used to assess differences in the categorized groups. Within- and between-group comparisons from baseline to the last follow-up were conducted using repeated-measures ANOVA to assess main effects and interactions. The *post hoc* comparison *P*-values were adjusted by Bonferroni correction.

Univariable and multivariable logistic regressions were performed to explore the impact of baseline, clinical, treatment, and lesion characteristics on the two groups. We selected variables for the multivariable logistic regression based on the baseline difference between the two groups. We did not include lesion characteristics in the multivariable model as this was influenced by intraoperative tremor improvement. However, as lesion size and DRTT overlap have been suggested as a reason for tremor relapse, we performed a *post hoc* sensitivity analysis including these variables in a linear regression model, which was favoured to avoid overfitting. In this model, the dependent variable was the HTS at the last follow-up. All statistical analyses were conducted using IBM SPSS Statistics (version 29.0.0.0).

## Results

Pre-and post-operative videos were available in 15 out of 17 patients for blinded tremor analysis. The median (ICR) follow-up period was 24 months (3–36). Two patients were lost to follow-up due to their geographical location and were not included in the blinded tremor analysis. All patients were male with a mean (SD) age of 71.5 (± 6.3) years and a median disease duration of 6 years (6–8) prior to treatment. The cohort mean baseline UPDRS-III was 38.2 (± 13.4) with a LEDD of 673.7 mg (± 375.6). All other patient demographics are reported in Table 1.

**Table 1:** Baseline demographics and clinical characteristics

Variable	All patients (*n* = 15)	Responders (*n* = 7)	Suboptimal Responders (*n* = 8)	Difference ‘R’ & ‘SR’ (*P* value)
Age, mean (SD), yrs	71.5 (6.3)	69.4 (6.5)	73.4 (6.0)	0.243
Disease duration, median (IQR), yrs	6 (6–8)	8 (5–9)	4.5 (3–6)	0.221
Medications failed, median (IQR)	3 (3–4)	3 (3–4)	3 (2.5–3.5)	0.752
Follow-up, median (ICR), mo	24 (3–36)	24 (6–36)	18 (3–30)	0.767
SDR, mean (SD)	0.43 (0.07)	0.42 (0.10)	0.44 (0.33)	0.759
LEDD, mean (SD), mg	673.7 (375.6)	833 (442.8)	534.4 (318.6)	0.129
UPDRS-III, mean (SD)	38.2 (13.4)	40.1 (16.5)	36.5 (11.0)	0.626
HTS, median (IQR)	11 (9.5–14.5)	11 (8–14.5)	12.3 (10.5–16.5)	0.523
Rest tremor UL, median (IQR)	4.0 (4.0–4.0)	4.0 (4.0–4.0)	4.0 (4.0–4.0)	0.350
Postural tremor UL, median (IQR)	4.0 (3.0–4.0)	4.0 (4.0–4.0)	4.0 (2.3–4.0)	0.373
Kinetic tremor UL, median (IQR)	0.5 (0.5–1.5)	0.5 (0–1.5)	0.8 (0.5–0 1.3)	0.401
HuTS, median (IQR)	5 (0.5–7.5)	2.5 (.5–7.5)	5 (0.8–7.5)	0.816
PDQ39, median (IQR)	22 (17–46)	22 (12–46)	25 (19–50)	0.728

HTS, Hand Tremor Score; HuTS, Hand untreated Tremor Score; LEDD, Levodopa Equivalent Daily Dose; PDQ39, Parkinson Disease Questionnaire 39; UL, Upper Limb, UPDRS-III, Unified Parkinson's Disease Rating Scale III; R, Responders; SDR, Skull Density Ratio; SR, Suboptimal Responders.

Across the entire cohort, baseline median HTS was 11.0 (9.5–14.5), improving to 6.0 (1–13.5) post treatment (*P* = 0.098). At the last follow-up, seven patients had greater than or equal to 50% improvement in HTS (R), while eight patients had less than 50% improvement (SR). The R group was younger at baseline, had a longer disease duration, a higher baseline LEDD, and had less tremor in the untreated hemibody (Table 1). The median follow-up time was similar for both groups (R: 24 (6–36); SR: 18 (3–30), *P* = 0.767), as were the other baseline demographic characteristics (Table 1).

At the final follow-up, the median HTS in the R group was 1 (0.5–2.5), while in the SR group, it was 13.3 (7.0–25) (Table 2). At baseline, both R and SR groups primarily experienced upper limb rest and postural tremor with minimal kinetic tremor (Table 1). The treatment effect on upper limb tremor in the R group was mainly seen in the rest and postural tremor subitems (Table 2). Although kinetic tremor was also significantly different between the two groups at the last follow-up (R: 0 (0–0); SR: 1 (0.3–3.5); *P* = 0.033); this difference cannot solely be attributed to a treatment effect, as kinetic tremor worsened in the SR group after treatment (Table 2).

**Table 2:** Clinical characteristics baseline and last follow-up

	Variable	Baseline	Last Follow-up	Difference Baseline & Last Follow-up (*P* value)	Difference ‘R’ & ‘SR’ at Last Follow-up (*P* value)
All Patients	HTS, median (IQR)	11 (9.5–14.5)	6 (1–13.5)	0.098	
*n* = 15	Rest Tremor UL, median (IQR)	4.0 (4.0–4.0)	2.5 (0–4)	0.008	
	Postural Tremor UL, median (IQR)	4.0 (3.0–4.0)	1.0 (0–4)	0.016	
	Kinetic Tremor UL, median (IQR)	0.5 (0.5–1.5)	0 (0–1)	0.876	
	HuTS, median (IQR)	5 (0.5–7.5)	5.5 (0.5–9.0)	0.774	
	PDQ39, median (IQR)	22 (17–46)	34 (23–49)	0.486	
	UPDRS-III, mean (SD)	38.2 (13.4)	34.6 (15.0)	0.510	
	LEDD, mean (SD), mg	673.7 (375.6)	741.0 (383.4)	0.637	
Responders					
*n* = 7	HTS, median (IQR)	11 (8–14.5)	1.0 (0.5–2.5)	0.010	0.002
	Rest Tremor UL, median (IQR)	4.0 (4.0–4.0)	0 (0–1.0)	<0.001	<0.001
	Postural Tremor UL, median (IQR)	4.0 (4.0–4.0)	0.5 (0–1.0)	<0.001	0.002
	Kinetic Tremor UL, median (IQR)	0.5 (0–1.5)	0 (0–0)	0.268	0.033
	HuTS, median (IQR)	2.5 (0.5–7.5)	0.5 (0.5–1.0)	0.293	0.004
	PDQ39, median (IQR)	22 (12–46)	28 (12–42)	0.950	0.175
	UPDRS-III, mean (SD)	40.1 (16.5)	31.7 (17.0)	0.162	0.515
	LEDD, mean (SD), mg	833 (442.8)	978.4 (406.0)	0.043	0.024
Suboptimal Responders					
*n* = 8	HTS, median (IQR)	12.3 (10.5–16.5)	13.3 (7.0–25.9)	0.341	
	Rest Tremor UL, median (IQR)	4.0 (4.0–4.0)	4.0 (3.25–4.0)	0.539	
	Postural Tremor UL, median (IQR)	4.0 (2.3–4.0)	3.5 (1.3–4.0)	0.452	
	Kinetic Tremor UL, median (IQR)	0.8 (0.5–1.4)	1.0 (0.3–3.5)	0.086	
	HuTS, median (IQR)	5 (0.8–7.5)	7.5 (5.6–11.4)	0.064	
	PDQ39, median (IQR)	25 (19–50)	40.5 (26.3–53.5)	0.211	
	UPDRS-III, mean (SD)	36.5 (11.0)	37.2 (14.6)	0.898	
	LEDD, mean (SD), mg	534.4 (318.6)	533.3 (261.0)	0.985	

HTS, Hand Tremor Score; HuTS, Hand untreated Tremor Score; LEDD, Levodopa Equivalent Daily Dose; PDQ39, Parkinson Disease Questionnaire 39; UL, Upper Limb; UPDRS--III, Unified Parkinson's Disease Rating Scale III; R, Responders; SR, Suboptimal Responders.

**Figure 1:**
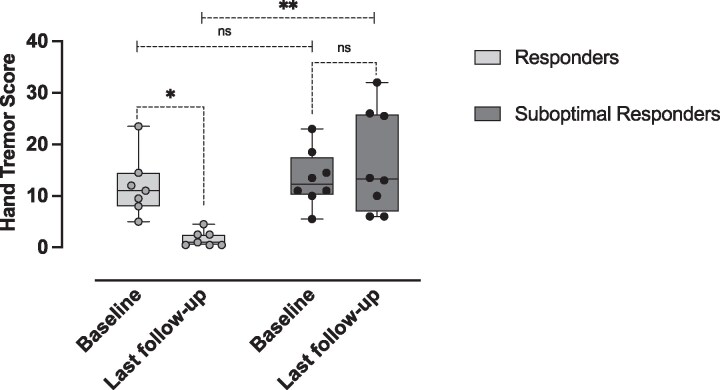
**Hand tremor score.** Whisker plots overlayed with individual patient’s data of Hand Tremor Score (HTS) at baseline and ‘last follow-up’ after treatment with a unilateral High-Intensity Focused Ultrasound (HiFUS) Thalamotomy. Patients grouped according to the degree of tremor improvement at the last follow-up, ≥ 50% ‘responders’ and < 50% ‘suboptimal responders’. **P < 0.01, *P < 0.05 (repeated-measures ANOVA, *post hoc* comparisons were adjusted by Bonferroni correction). N: R = 7, SR = 8.

R had a higher baseline LEDD; a 17% increase in LEDD was also observed in this group after treatment (*P* = 0.043), which was not seen in the SR (Table 2). Further, at the final follow-up, the R group LEDD was 83% greater than that in the SR group (R: 978.4 mg ±406.0, SR: 533.3 mg ±261.0; *P* = 0.024). Furthermore, tremor in the ipsilateral hemibody (HuTS), generally considered the untreated side, improved following treatment in the R group, while in the SR group the tremor severity increased. This correlated to a larger and significant difference in the HuTS between the two groups at the last follow-up (R: 0.5 (0.5–1.0), SR: 7.5 (5.6–11.4), *P* = 0.004), (Table 2, Fig. 1).

A median of 9 (8–11) sonications and 4 (3–4) therapeutic sonications were required to achieve tremor control at the end of the HiFUS procedure, which was similar between the two groups (Table 3). However, there was a trend for larger lesion parameters in the SR group with a median total lesion volume of 157.5mm^3^ (113.4–215) versus 119.7mm^3^ (111.6–237.6) and median core lesion volume of 17.9mm^3^ (15.0–20.9) versus 9.6mm^3^ (7.5–24). The cohort mean AC–PC distance was 25.4 mm (±1.45) with the mean lesion coordinates: X = 13.9 mm (±0.82), Y = 7.0 mm (±0.48) and Z = 0.8 mm (±0.65). Lesions in the R group (14.3 mm ±0.69 mm) were more lateral than those in the SR group (13.5 mm ±0.81) (*P* = 0.082). However, there was no difference in the anterior-posterior or superior-inferior axis (Table 3).

**Table 3:** Treatment parameters

Variable	All patients (*n* = 15)	Responders (*n* = 7)	Suboptimal Responders (*n* = 8)	Difference ‘R’ & ‘SR’ (*P* value)
Sonication’s, median (IQR), No.	9 (8–11)	10 (8–12)	9 (8–10)	0.338
Sonification’s > 53°, median (IQR), No.	4 (3–4)	4 (3–4)	4 (4–4)	0.128
AC–PC distance, mean (SD), mm	25.4 (1.45)	25.7 (1.26)	25.2 (1.64)	0.550
X, VIM, mean (SD), mm	13.9 (0.82)	14.3 (0.69)	13.5 (0.81)	0.082
Y, VIM, mean (SD), mm	7.0 (0.48)	7.1 (0.31)	6.9 (0.61)	0.611
Z, VIM, mean (SD), mm	0.8 (0.65)	0.9 (0.73)	0.7 (0.60)	0.565
Y relative to AC–PC, mean (SD), %	27.5 (1.26)	27.6 (1.29)	27.5 (1.32)	0.923
Lesion volume, median (IQR), mm^3^	155.4 (111.6–228.9)	119.7 (111.6–237.6)	157.5 (113.4–215)	0.908
Core Volume, median (IQR), mm^3^	16.8 (9.6–22.5)	9.6 (7.5–24.9)	17.9 (15.0–20.9)	0.355
PSA lesions, No. (%)	5 (33)	1 (14.3)	4 (50)	0.143
DRTT-lesion overlap, mean (SD), %	83.5 (72.5–84.8)	82.6 (64.3–83.7)	84.8 (72.5–92.7)	0.224
*n* = 13		*n* = 6	*n* = 7	
DRTT-core overlap, mean (SD), %	40.3 (28.5–41.7)	27.1 (13.8–39.3)	41.7 (40.5–48.8)	0.010
*n* = 13		*n* = 6	*n* = 7	

AC, Anterior Commissure; DRTT, Dentatorubrothalamic Tract; PC, Posterior Commissure; PSA, Posterior Subthalamic Area; R, Responders; SR, Suboptimal Responders; VIM, Ventral Intermedius Nucleus.

**Figure 2:**
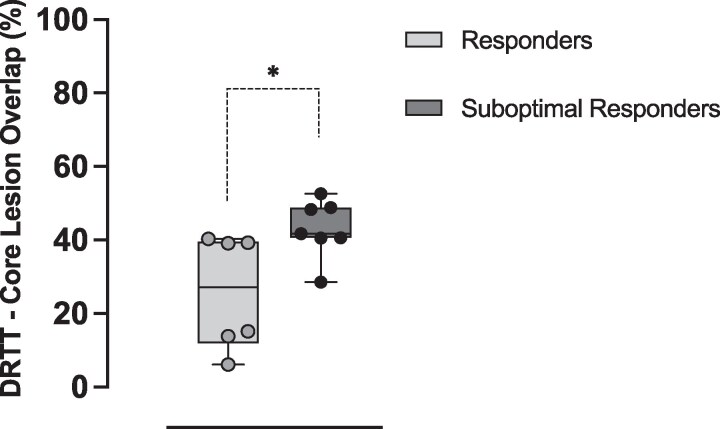
**Dentatorubrothalamic tract-core ablation overlap.** Whisker plots overlayed with individual patient’s data representing the percentage of dentatorubrothalamic tract-core ablation overlap in ‘responders’ and ‘suboptimal responders’. *P < 0.05 (Student *t*-test) N: R = 6, SR = 7.

Data from 13 patients (R = 6, SR = 7) were available to calculate DRTT volume overlaps. There was no difference in the DRTT total lesion overlap between the two groups (R: 82.6% (64.3–83.7); SR 84.8% (72.5–92.7), *P* = 0.224). However, there was a difference in the DRTT-core lesion overlap, greater in the SR group (R: 27.1% (13.8–39.3%), SR: 41.7% (40.5–48.8%), *P* = 0.010) (Table 3, Fig. 2).

Supplementary Table 1 shows the analysis of the logistic regressions, with R and SR as the response variables. No baseline or treatment parameters predicted the two outcomes. These included the observed baseline group differences: age, disease duration, LEDD and HuTS. Treatment parameters were not included in the multivariable logistic regression due to the risk of overfitting. In the *post hoc* multivariable linear regression, lesion and core volumes, and, DRTT-lesion and core overlap were included alongside baseline HTS and group differences (age, disease duration, LEDD and HuTS). This showed that the DRTT-core overlap was associated with a higher HTS at the last follow-up (β 1.06, *P* = 0.008) (Table 4). This aligns with the observation that the SR group had greater DRTT-core overlap. Unexpectedly, a higher baseline HuTS was associated with a lower HTS at the last follow-up HTS (β −2.24, *P* = 0.016) (Table 4).

**Table 4:** Multivariable linear regression, HTS last follow-up

Variable	Beta coefficient	95% CI	*P*-value
Age	−0.14	−0.93 to 0.64	0.614
Disease duration	−0.37	−2.33 to 1.59	0.586
LEDD	−0.001	−0.12 to 0.01	0.762
Baseline HTS	−0.14	−1.07 to 0.87	0.766
HuTS	−2.24	−3.70 to −0.78	0.016
Lesion volume	−0.06	−0.20 to 0.08	0.264
Core volume	−0.10	−1.64 to 1.44	0.853
DRTT-lesion overlap	−0.29	−0.74 to 0.16	0.133
DRTT-core overlap	1.06	0.54 to 1.58	0.008

DRTT, Dentatorubrothalamic Tract; HTS, Hand Tremor Score; HuTS, Hand untreated Tremor Score; LEDD, Levodopa Equivalent Daily Dose; UPDRS-III, Unified Parkinson's Disease Rating Scale III.

Adverse events in this patient population were similar in frequency and severity to other studies from our centre involving patients with different tremor syndromes (Supplementary Table 2).^10,14^ A total of 87% of all individuals experienced at least one side effect, persisting in 20% of patients in the long-term (1 year). The most common issue was gait disturbance, affecting both groups equally. In the first month, 80% of the cohort experienced gait disturbance, mild in 67% and moderate in 33% of cases. By months 3–6, gait disturbance was affecting 29% of the cohort, all of which were of mild severity. One individual experienced moderate weakness of the contralateral lower limb, which improved to mild severity at 6 months and had resolved at 12 months.

## Discussion

This is the first post-marketing blinded tremor evaluation of patients with TdPD who have undergone a unilateral HiFUS thalamotomy. Further, we have correlated tremor outcomes with ablation volumes and percentage of DRTT overlap. In our cohort, we did not observe an overall improvement in tremor scores over a median follow-up period of 24 months. However, approximately half of the patients obtained a greater than 50% tremor improvement at the final follow-up. The most important and interesting finding of the study is the demonstration that the DRTT-core overlap was greater in the suboptimal responders when compared to responders and that the DRTT-core overlap was associated with a greater HTS at the final follow-up. In view of the knowledge that clinical outcomes in ET have been positively correlated with the DRTT-lesion overlap in a number of studies,^14-16^ these findings provide further evidence that the most efficacious HiFUS tremor target likely differs between ET and TdPD.^12^

Our clinical outcomes are in contrast to most larger post-marketing HiFUS thalamotomy studies in TdPD, which generally report durable medium to long-term tremor control.^5-7,28,29^ However, there is a growing literature recognizing the risk of tremor relapse in TdPD after HiFUS thalamotomy.^9,10,30,31^ In *Braccia and colleagues* cohort, tremor relapse was reported in 23% of patients at 6 months, which was exclusively observed in the first month after treatment. This was associated with a younger age at surgery and smaller lesion volumes, a contrasting finding to suboptimal responders in our cohort, which were older and had larger lesion volumes. There is an important difference in the two cohorts; we studied an older cohort with a longer disease duration and higher LEDD at baseline. Further, despite *Braccia and colleagues* much larger sample size, close to 30% of patients treated during the study period remain unaccounted, and in our experience generally represent patients with suboptimal outcomes. While in the medium-term, *Paschen and colleagues* comparison of HiFUS targeting the Vim and STN, close to 50% of Vim-HiFUS patients were considered insufficient or non-responders at 12 months, a decline that was observed between 4 and 12 months.^11^

Despite failure to demonstrate improvement in tremor scores over a median follow-up of 24 months, unilateral HiFUS thalamotomy at our centre did result in sustained tremor improvement in approximately half of the cohort (7/15) in this timeframe. An arbitrary cut-off of 50% tremor improvement in HTS at the last follow-up was chosen to divide ‘responders’ and ‘suboptimal responders’. This is aligned with *Bond and colleagues’* definition of ‘success’ in the open-label phase of their original trial, and in comparison to 55–65% of patients obtaining ‘success’, depending on the method of statistical analysis, our results are comparable, considering the extended follow-up period.^2^ In our suboptimal responders, four patients went on to have deep brain stimulation (DBS), two were due to tremor relapse within 3 months and two due to tremor relapse at 12 and 24 months in the context of progressive disease, spread of tremor to contralateral hemibody and worsening bradykinesia/rigidity.^10,30^ A reflection of the variable timing of tremor relapses in this population after a unilateral HiFUS thalamotomy.

The majority of previous HiFUS thalamotomy studies have reported unchanged LEDD in the short and medium-term.^5-7,9,32^ Authors have even suggested that the treatment can prevent future increases in Levodopa.^32^ However, in the only previous long-term follow-up series of TdPD, an increase of LEDD emerged at 2 years, and was approximately 60% greater than baseline at 3 years.^6^ In our study, responders had a higher baseline LEDD in the context of a longer disease duration, but importantly, in this group, a 17% increase in LEDD was observed post treatment, and at the last follow-up, there was an 83% greater LEDD in responders compared to suboptimal responders (Table 3). It is likely that the higher LEDD in responders, particularly at the last follow-up, contributed to some degree to the superior outcome, though given the ‘medication refractoriness’ of the tremor at baseline, it is unlikely to be the sole cause. A major limitation of our study is the lack of *off* assessment, meaning the treatment effect alone of HiFUS thalamotomy cannot reliably be determined. Nor are we able to comment on the percentage improvement in tremor with levodopa pre- or post-treatment. It is possible that a HiFUS thalamotomy may make levodopa more effective in treating tremor in PD, a possible mechanism in responders that was not utilized or present in suboptimal responders, which will need to be explored through the levodopa challenge in future HiFUS thalamotomy studies.

Exploration of baseline patient, tremor and disease factors showed that higher baseline tremor in the untreated side was associated with a lower HTS at the last follow-up. This is an unexpected finding and might be due to the observed improvement in the HuTS amongst responders, potentially an ipsilateral treatment effect or a response to the increase in LEDD at the final follow-up. A small subset of patients obtain improvement in the ipsilateral tremor following unilateral HiFUS thalamotomy, presumably from either lesioning of the non-decussating DRTT^33^ or simply a reduction in tremor transmission from the treated to the untreated side.^34,35^ Caution should be taken when interpreting this association due to the small sample size, *post hoc* analysis, and that the result may represent statistical chance.

Authors have hypothesized that tremor relapse following unilateral HiFUS thalamotomy in TdPD may be attributed to smaller lesion size and insufficient overlap with the DRTT.^7,13,36^ Early studies that supported this theory used relatively simple lesion topography analyses.^13^ A more recent study by *Braccia and colleagues* supports aspects of this hypothesis, demonstrating that smaller lesion sizes were associated with relapse in the first 6 months. However, in our cohort, suboptimal responders tended to have larger lesions. While all patients experienced tremor freedom by the end of the HiFUS procedure, poor intraoperative response to initial sonication necessitated a target shift to the PSA in half of the suboptimal responders and possible further spot shifts within the Vim (though this was not captured in our dataset). These shifts ultimately contribute to larger lesions in suboptimal responders, and when considered with the greater DRTT-core overlap in these individuals, it is likely evidence of a subpopulation that have resistant tremor prone to relapse.

There are numerous lines of evidence that implicate both the BG and the DRTT in PD tremor genesis.^19^ Electrophysiological studies have identified neuronal cells firing at tremor frequencies in the subthalamic nucleus (STN),^37^ globus pallidus^38^ and thalamus.^39^ Further, tremor benefit is seen following individual lesioning of each of these targets in PD.^40^ Inferences have been made as to the origin of PD tremor based on the synchronicity of BG and thalamic cells with peripheral electromyography recordings.^41,42^ The variable coherence of pallidal neurons with tremor is evidence to many that the thalamus is the driving force behind tremor in PD. Many models have been put forward to explain the pathogenesis of tremor in PD; though the most widely accepted is the integrated ‘dimmer-switch’ model constructed by *Helmich and colleagues*.^19^ Conceptually, they propose that the BG triggers the tremor system, akin to a ‘light switch,’ while the DRTT contributes by modulating the tremor intensity as would a ‘light dimmer’. However, this mechanism should theoretically result in a positive correlation with tremor outcomes and the DRTT lesion overlap, rather than fact that the opposite was observed in our cohort.

A number of groups, including ours, have localized the optimal site for a unilateral HiFUS thalamotomy in ET to the posterior region of the VIM when targeting at the level of the AC–PC, which has correlated to the proportion of DRTT lesion overlap.^14-17^ Despite the pathophysiological differences, this ‘traditional’ stereotactic tremor target has generally been adopted for TdPD. To our knowledge, only one study has demonstrated a similar correlation in TdPD, though the measurement used for this analysis was an arbitrary 50% transection of the DRTT. Our study demonstrates a contrasting finding, that the proportion of DRTT lesion overlap in TdPD was not associated with responders, and in fact, suboptimal responders had a significantly greater core volume DRTT overlap. Recently, probabilistic mapping of HiFUS lesions, using data from multiple sites, has also provided evidence that the most effective lesioning site in TdPD differs from ET, at the Vim-ventralis oralis posterior (Vop) junction.^12^ While the *Obeso* group in Madrid has used lesion topography to demonstrate that larger Vim lesions extending into the dorsum of the STN are associated with sustained tremor improvement in TdPD.^43^ However, much of probabilistic mapping is spatially limited, where a ‘sweet spot’ is identified around the intended target. In a recent staged bilateral HiFUS ET cohort, the probabilistic lesion maps were spatially extended in view of the differing targeting methods between sides. It was identified that the optimal Vim target differed depending on the targeting method implemented (dorsal versus ventral).^44^ Similar spatial differences likely account for the distinct ‘sweet spot’ differences in these two TdPD HiFUS studies. However, both methods have the potential to disrupt the BG circuits in addition to the DRTT: the first method via pallidothalamic fibres, while the second through subthalamopallidal fibres—both of which align with current pathophysiological models of TdPD.

In our cohort, responders had a more lateral lesion placement without any differences in the anterior-posterior or superior-inferior lesion topography. The homunculus of the motor thalamus, assumed primarily from primate studies, suggests the leg is located most laterally, while the arm and face are found progressively medial.^45^ It is possible that the more medial lesions in suboptimal responders were too medial and missed the critical somatotopic hand region. While addition disruption to the BG circuits in responders is unlikely, given the lack of difference in lesion topography in the anterior-posterior or superior-inferior planes. However, the higher LEDD at baseline and last follow-up, in conjunction with the lack of correlation between DRTT lesion overlap and clinical outcome, again highlights the importance of the BG circuits in both the pathophysiology and treatment of tremor in PD. A lack of disease specific targeting in HiFUS has been proposed for the variable outcomes in TdPD,^12^ and our ‘traditional’ Vim targeting is likely in part to have contributed to tremor relapse in suboptimal responders.^30^ It is possible that in addition to our posterior VIM lesions covering the DRTT, further lesioning to the BG circuit, depending on the targeting approach, either at the Vim/Vop border or dorsal STN, would improve the outcomes, and potentially prevent tremor relapse. While escalation in levodopa to a potentially more dopamine-responsive tremor might also have a similar outcome.

The frequency of adverse events (AEs) in this study, particularly gait disturbance, is higher than what is typically reported in the literature. This discrepancy may be partly due to the younger TdPD cohorts in most previous studies, who may be less susceptible to such events,^2,46^ or simply due to methodological biases. One example of the latter is when gait disturbance is categorized into two AEs (gait instability or ataxia), and the true occurrence of the AE is minimized.^46^ When our study is compared to an age-matched cohort treated at two international centres, the AE profiles show notable similarities.^11^ Though it is possible that the additional lesion targeting the PSA in a subset of patients contributed to the higher incidence of gait disturbances.^14^ No significant difference in gait disturbance was observed between responders and those with a suboptimal response, even though 50% of the latter group required an additional PSA treatment. Importantly, even when compared to younger TdPD cohorts, the rates of moderate or serious AEs, those most likely to affect quality of life, are comparable or more favourable in our study. Nevertheless, the AE profile of HiFUS ablation in TdPD will need to be continually re-analysed as the optimal HiFUS targeting method and location is further refined.

It is difficult to reconcile our results given the historic outcomes observed after both radiofrequency (RF) lesioning and DBS of the thalamus in TdPD.^47^ However, RF thalamotomy usually involves larger ablation volumes, from multiple lesions, extending along the electrode tract,^48^ potentially disrupting additional circuits, such as the pallidothalamic or subthalamopallidal tract. While DBS of the thalamus is known to have effects beyond the primary site of stimulation, with reduction of metabolic correlates of tremor, having been demonstrated in the BG and motor cortex during ‘On-Off’ functional imaging studies.^49^ Both are reasons for caution when attempting to extrapolate outcomes for stereotactic targets between different modalities of treatment, especially given the explosion of HiFUS over the last decade.^50^

Our study has several limitations, including the small sample size, lack of female participants, an arbitrary 50% cut-off to be considered a ‘responder’, assessment in the *on-medication* state, and a high number of patients that failed to attend the pre-defined clinical assessment points. We have attempted to account for the latter using the time point ‘last follow-up’, to minimize attrition bias and the observation that individuals who have had a suboptimal outcome to HiFUS are unlikely to return for more than one post-operative research assessment. Further, some patients received lesions in the PSA, performed to address residual intraprocedural tremor, but this was almost exclusively associated with suboptimal responders and provides further support that tremor relapse in TdPD after HiFUS thalamotomy is not solely due to lesion topography, or more specifically, the extent of DRTT ablation. Finally, caution should be taken when interpreting the DRTT results in isolation, given there is still no universally accepted method to reconstruct the DRTT via diffuse tensor imaging.^51^

In summary, we have found that unilateral HiFUS thalamotomy in TdPD resulted in sustained tremor reduction in approximately 50% of patients. These patients were younger, with a longer disease duration, had less tremor in the untreated hemibody and had a higher LEDD at the last follow-up. While tremor improvement did not correlate with DRTT ablation overlap or lesion size, providing indirect evidence that the most efficacious HiFUS tremor target differs between ET and TdPD. Further prospective studies with careful neurological evaluation and follow-up are required before unilateral HiFUS thalamotomy can be recommended for TdPD on a routine clinical basis.^4^

## Supplementary Material

fcaf303_Supplementary_Data

## Data Availability

The data that support the findings of this study are available on request from the corresponding author. The data are not publicly available due to privacy and ethical restrictions. New software or algorithms, including in-house scripts or programmes, were not used during the study.
