# Supplementary material for: Unilateral focused ultrasound thalamotomy for tremor-dominant Parkinson’s disease: blinded evaluation and imaging correlation
Source: Brain Commun. 2025 Aug 19;7(4):fcaf303. doi: 10.1093/braincomms/fcaf303 (PMC12392903; doi:10.1093/braincomms/fcaf303)
Supplement: fcaf303_Supplementary_Data [file fcaf303_supplementary_data.pdf]

Supplementary Table 1: Logistic regression

Univariable	Odds Ratio	P value	Multivariable	P value
Age, yrs	0.89 (0.74 – 1.07)	0.234	0.92 (0.76 – 1.12)	0.396
Disease duration, yrs	0.98 (0.85 – 1.12)	0.733	0.97 (0.83 – 1.14)	0.729
Medications failed	1.03 (0.40 – 2.68)	0.955		
Follow-up, mo	1.01 (0.94 – 1.09)	0.744		
LEDD, mg	1.00 (1.00 – 1.00)	0.168	1.00 (1.00 – 1.01)	0.230
UPDRS III	1.02 (0.94 – 1.11)	0.597		
HTS	0.95 (0.78 – 1.16)	0.602		
HuTS	0.92 (0.68 – 1.24)	0.566	1.03 (0.72 – 1.48)	0.861
Action tremor	0.45 (0.06 – 3.12)	0.416		
PSA lesions	0.17 (0.01 – 2.09)	0.165		
Sonication's	1.2 (0.79 – 1.83)	0.394		
Sonication's > 53°	0.25 (0.02 – 2.43)	0.229		
Lesion volume, mm^3	1.00 (0.98 – 1.01)	0.804		
Core volume, mm^3	0.91 (0.77 – 1.08)	0.294		
DRTT-lesion overlap	0.93 (0.83 – 1.05)	0.423		
DRTT-core overlap	0.87 (0.74 – 1.03)	0.103		
AC-PC distance, mm^3	0.78 (0.37 – 1.64)	0.519		
VIM x, mm	0.21 (0.03 – 1.44)	0.113		
VIM y, mm	0.53 (0.06 – 4.99)	0.582		
VIM z, mm	0.59 (0.11 – 3.16)	0.538		
VIM y - relative to PC, %	0.96 (0.42 – 2.20)	0.916		

Abbreviations: AC, Anterior Commissure; DRTT, Dentatorubrothalamic Tract; HTS, Hand Tremor Score; HuTS, Hand untreated Tremor Score; LEDD, Levodopa Equivalent Daily Dose; PDQ39, Parkinson Disease Questionnaire 39; PC, Posterior Commissure; PSA, Posterior Subthalamic Area; UPDRS III, Unified Parkinson's Disease Rating Scale III; VIM, Ventral Intermedius Nucleus.

Supplementary Table 2: Adverse Events

	Intraprocedural (n = 15)	Short-term (n = 15)	Medium-term (n = 14)	Long-term (n = 10)
Any AE, N (%)		13 (87)	4 (29)	2 (20)
Gait Disturbance		12 (80)	4 (29)	2 (20)
Grade 1		8	4	2
Grade 2		4	-	-
Grade 3		-	-	-
Fatigue		2 (13)	-	-
Dysmetria		1 (7)	-	-
Hiccups		1 (7)	-	-
Weakness		1 (7)	1 (7)	-
Taste		1 (7)	-	-
Any AE, N (%)	6 (40)			
Vertigo	8 (53)			
Pain/Headache	4 (27)			
Nausea	2 (13)			
Others	0			
